# CAM-UNet: A Novel Water Environment Perception Method Integrating CoAtNet Structure

**DOI:** 10.3390/s25226963

**Published:** 2025-11-14

**Authors:** Xingyi Gao, Jie Liu, Yanyi Liu, Yin Wu

**Affiliations:** 1 College of Information Science and Technology & Artificial Intelligence, Nanjing Forestry University, Nanjing 210037, China; nfugxy@njfu.edu.cn (X.G.); yyliu@njfu.edu.cn (Y.L.); 2 Qingdao Guoshi Intelligent Equipment Technology Co., Ltd., Qingdao 266000, China; jliu05328@gmail.com

**Keywords:** deep learning, CoAtNet, semantic segmentation, image processing, unmanned surface vessel

## Abstract

Accurate segmentation of navigable waters and obstacles is critical for unmanned surface vessel navigation yet remains challenging in real aquatic environments characterized by complex water textures and blurred boundaries. Current models often struggle to simultaneously capture long-range contextual dependencies and fine spatial details, frequently leading to fragmented segmentation results. In order to resolve these issues, we present a novel segmentation model based on the CoAtNet architecture. Our framework employs an enhanced convolutional attention encoder, where a Fused Mobile Inverted Bottleneck Convolution (Fused-MBConv) module refines boundary features while a Convolutional Block Attention Module (CBAM) enhances feature awareness. The model incorporates a Bi-level Former (BiFormer) to enable collaborative modeling of global and local features, complemented by a Multi-scale Attention Aggregation (MSAA) module that effectively captures contextual information across different scales. The decoder, based on U-Net, restores spatial resolution gradually through skip connections and upsampling. In our experiments, the model achieves 95.15% mIoU on a self-collected dataset and 98.48% on the public MaSTr1325 dataset, outperforming DeepLabV3+, SeaFormer, and WaSRNet. These results show the model’s ability to effectively interpret complex aquatic environments for autonomous navigation.

## 1. Introduction

In recent years, with the continuous advancement of artificial intelligence technology, unmanned systems such as drones and self-driving cars have received widespread attention. Unmanned surface vehicles (USVs) are an important branch of this field and have shown broad application prospects in the fields of ocean surveys, environmental monitoring, water rescue and resource development [1]. In order to achieve autonomous navigation and mission planning, USVs need to have efficient environmental perception capabilities. Among them, accurately segmenting the traversable area and obstacles on the water surface is a key task in its visual perception system, which affects the safety and execution efficiency of the navigation path [2].

The environmental perception of USVs is the basis for their autonomous navigation and mission execution. They usually collect visible light images by carrying visual sensors and perform semantic segmentation of the water surface, land and obstacles [3]. Common sensors include monocular or binocular RGB cameras, panoramic cameras, infrared imaging equipment, LiDAR, radar and multispectral/hyperspectral sensors. Among them, visual cameras have the advantages of low cost and rich information and are the most widely used perception devices; infrared sensors can assist in identifying targets at night or in low-light environments; lidar can provide accurate three-dimensional spatial information, which is suitable for structural mapping and obstacle detection; and radar has strong penetration and can maintain stable perception under adverse weather conditions [4]. Current image segmentation methods mainly include traditional image processing and deep learning.

Traditional image segmentation methods mainly include edge detection, threshold segmentation, active contour model and region growing, etc. [5]. These methods have low computing power and data requirements and can run efficiently on low-power edge devices, but they are difficult to handle complex scenes. Edge detection methods (e.g., Canny) often produce fragmented boundaries, while thresholding techniques require manual parameter tuning; active contour models are highly sensitive to initialization and image intensity variations [6]; region growing can improve local accuracy but suffers from high computational cost and sensitivity to seed selection [7]. To address these issues, Peng et al. [8] proposed combining the HSV color model with edge detection for shoreline extraction, though the method lacked robustness for irregular boundaries. Zheng et al. [9] integrated Otsu thresholding with region growing to enhance accuracy, but at the cost of increased computation.

Deep learning-based methods have been extensively applied to water surface segmentation [10,11,12,13], with Convolutional Neural Networks (CNNs) demonstrating strong capability in extracting local features. The adaptive scale network SANet proposed by Cui et al. [14] performed well in the segmentation task of the coastal area of Lianyungang, Jiangsu, but it has not been verified on data from other regions, which limits its applicability in a wider range of scenarios. Guo Yangang et al. [15] improved PSPNet by introducing transfer learning and attention mechanism to reduce the false detection rate, but the recognition of details in the fuzzy boundary area is still limited; Xiong Rui et al. [16] proposed DeepLabV3-CSPNet based on DeepLabV3, which improved the accuracy, but the stability is still insufficient under extreme lighting conditions. Zhang Liya et al. [17] proposed BEMSNet, which effectively improved the boundary recognition accuracy in the semantic segmentation of unmanned surface vehicle navigation images through the innovative design of boundary abstraction module and boundary enhancement module, However, it still has limitations, such as poor adaptability to extreme weather scenarios. On the other hand, the Transformer architecture has demonstrated superior semantic understanding performance due to its excellent global modeling capabilities. Representative models such as Swin Transformer [18] and SegFormer [19] have achieved leading results in multiple segmentation tasks. However, the Transformer model has large parameter scale, high computational resource consumption, complex deployment, and is prone to overfitting in small sample tasks. Its application in USV visual perception still faces challenges.

Based on the above problems, this paper proposes an improved semantic segmentation network CAM-UNet based on CoAtNet. The main contributions are as follows:

(1) We proposed CAM-UNet, an improved encoder–decoder network that combines convolutional structures and attention mechanisms to capture both local textures and global semantics. The model was specifically designed to handle water surface segmentation tasks, especially those involving complex boundaries or small targets.
(2) We implemented a multi-stage feature extraction framework based on the CoAtNet backbone, incorporating various modules to improve both local details and global context understanding. To handle challenging conditions such as surface ripples and strong reflections, we introduced a module between the encoder and decoder to improve multi-scale feature representation.
(3) We created and manually annotated a semantic segmentation dataset containing 4950 images covering seven types of water surfaces and six obstacle categories. The dataset included more than 10 representative scenarios and was used for both model training and performance evaluation.

## 2. Materials and Methods

### 2.1. Experimental Details

All experiments were conducted on a workstation equipped with an NVIDIA GeForce RTX 4070 GPU (12 GB VRAM) from ASUS (Taipei, China), an Intel Core i7-13700KF processor from Intel Corporation (Santa Clara, CA, USA), and 64 GB of memory. The model was implemented using the PyTorch 1.13.0 framework with Python 3.8 and accelerated by CUDA 11.6 and cuDNN 8.4. During training, the Adam optimizer was employed with an initial learning rate of 1 × 10^−4^ and a batch size of 8. The learning rate was gradually decayed using a cosine annealing strategy, and the model was trained for a total of 200 epochs. The experimental equipment configuration is shown in Table 1.

The loss functions used in this experiment are Cross Entropy Loss and Dice Loss. Since the foreground regions—especially obstacle classes—occupy relatively small areas in the dataset, while the background covers a much larger proportion, using a conventional Cross Entropy Loss (CE Loss) alone may lead to the positive samples being overwhelmed by the negative ones. Dice Loss is more sensitive to positive samples in the early stages of training and thus helps to better capture the foreground regions. However, it may suffer from loss saturation; therefore, it is combined with Cross Entropy Loss to achieve a balanced optimization.

### 2.2. Dataset Construction and Processing

The task of segmenting feasible water domains and obstacles involves high scene complexity and substantial visual interference, placing stringent requirements on the quality, diversity, and annotation precision of the training data. To ensure reliable performance in various environments, the dataset should cover multiple types of water bodies and scene settings. Additionally, a wide range of lighting conditions, weather patterns, and time of day should be taken into account to make the dataset content rich and diverse.

At the object level, the dataset should include various common surface structures as well as dynamic obstacles. These obstacles should vary in size, direction, material properties, and occlusion level. This diversity ensures that the dataset closely reflects the real-world challenges faced in navigable water environments. Additionally, the dataset should also contain a large number of pure water scenes, which will help the model better learn the navigable boundaries, surface textures, and shape changes, ultimately facilitating clearer classification during the segmentation process. Sampling.

Based on these requirements, this paper constructed a water surface image dataset suitable for this study through self-collection and online search. The final dataset contains a total of 4950 images, which are divided into a training set of 3465 images, a validation set of 990 images, and a test set of 495 images according to a 7:2:1 ratio.

**Figure 1:**
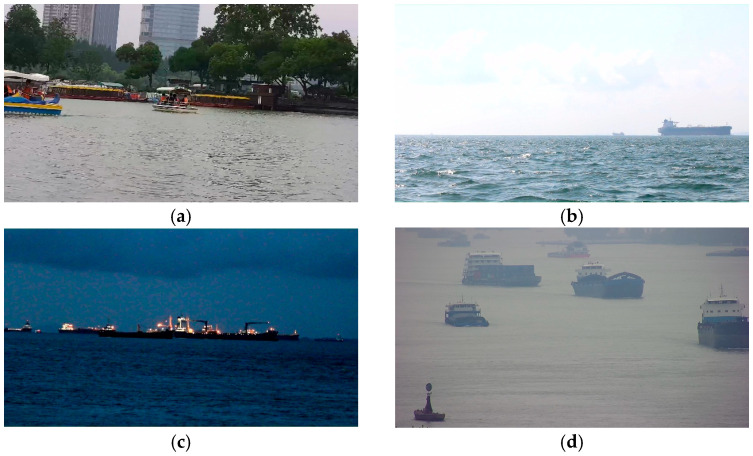
Examples of WSM USV data in the self-built dataset. (**a**) Daytime with clear sky; (**b**) daytime with rain; (**c**) evening with fog; (**d**) nighttime.

Image acquisition was conducted in several representative aquatic environments using high-definition RGB cameras mounted on an autonomous surface platform and fixed onshore equipment. The captured images cover various times of day and incorporate perspective variations and reflection disturbances in various natural environments. The captured images are shown in Figure 1, encompassing daytime, nighttime, sunny, rainy, and foggy conditions. Labels include feasible regions and obstacles.

**Figure 2:**
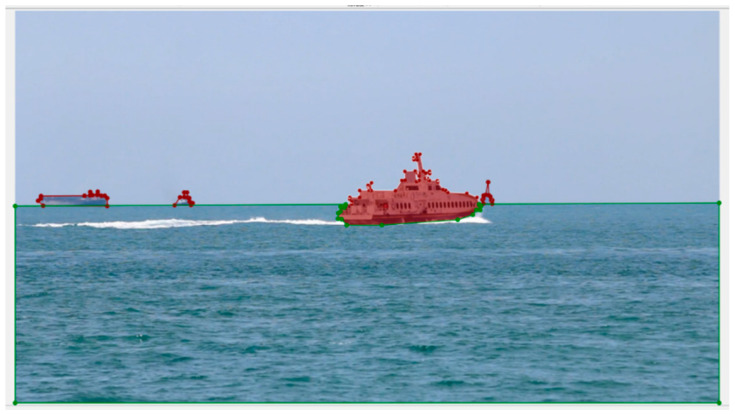
Example of annotation.

The original image was manually annotated frame by frame using Labelme 5.8.1 software. The polygonal outline of the target area was accurately drawn to generate the corresponding segmentation label. The annotation process is as follows. The example of annotation is shown in Figure 2.

**Figure 3:**
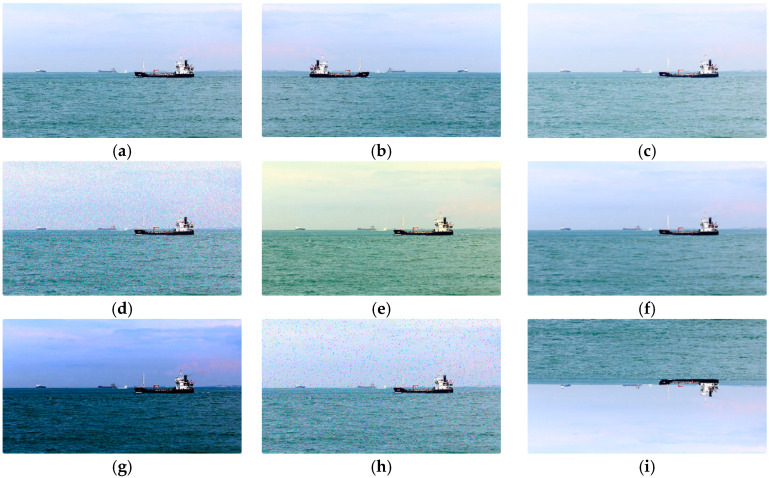
Examples of data augmentation. (**a**) Original image; (**b**) horizontal flip; (**c**) brightness adjustment; (**d**) Gaussian noise; (**e**) color jittering; (**f**) image blurring (**g**) contrast adjustment; (**h**) salt and pepper noise; (**i**) vertical flip.

To enhance the model’s generalization capabilities and adaptability to diverse environmental changes, this study performed data augmentation on the training set. Obstacle types and backgrounds in water scenes are complex and varied, and images may be subject to lighting variations, angle offsets, and object occlusions, all of which can affect model training. To this end, we employed common image augmentation techniques, including rotation, flipping, scaling, cropping, brightness adjustment, and salt-and-pepper noise. Figure 3 shows some of these data augmentation methods.

### 2.3. Methods

#### 2.3.1. CAM-UNet Overall Framework

**Figure 4:**
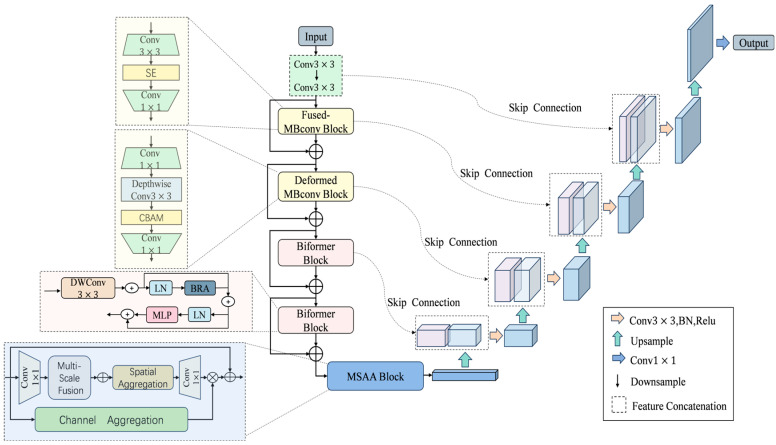
Overall framework diagram of CAM-UNet.

CAM-UNet is an improved encoder–decoder network architecture for water surface feasible domain and obstacle segmentation tasks. The encoder is structurally optimized based on CoAtNet and employs a multi-scale attention mechanism to improve segmentation accuracy. The decoder adopts a U-Net architecture. Figure 4 shows the overall framework of CAM-UNet.

In the encoder, the architecture is refined from CoAtNet and designed to perform hierarchical, progressive feature extraction. At the initial S0 stage, two successive 3×3 convolutional layers capture fundamental texture patterns, supplying abundant low-level cues to support higher-level semantic reasoning. The S1 stage incorporates the Fused-MBConv module, which enhances the representation of local textures and boundary structures while keeping computational costs low.

In the S2 stage, we use an improved MBConv block adapted from MobileNetV3, where the original SE attention mechanism is replaced by the CBAM mechanism. This change enables the model to more accurately focus on key areas under conditions such as complex water surface reflections, changing lighting, and deformable obstacles. As a result, the model’s ability to distinguish different features has been significantly enhanced.

For the deeper semantic stages S3 and S4, we employed the BiFormer [20] module, which features a bidirectional attention mechanism that can effectively capture local dependencies and long-distance context relationships. This enables the model to significantly enhance its ability to identify distant obstacles and large-scale scene layouts, while reducing the impact of background noise. The MSAA [21] module is located between the encoder and the decoder, and it can collect multi-scale context cues, thereby further improving the segmentation accuracy.

The decoder part employs the U-Net architecture, which gradually reconstructs high-resolution feature maps through upsampling and skip connections. It has excellent symmetry and strong feature restoration capabilities, providing robust performance and improving segmentation quality. Especially in complex environments, it can handle tasks that require high-precision outputs.

#### 2.3.2. CoAtNet Network Structure

Due to its local perception ability and inductive bias, CNN [22] has outstanding generalization ability and convergence speed during training and is suitable for small sample scenarios and low-latency tasks. However, the limitation of CNN is that its receptive field is limited, and it is difficult to capture long-distance dependencies. Compared with CNN, Transformer [23] achieves global modeling through the self-attention mechanism, which is suitable for modeling complex semantic relationships. However, it lacks local inductive priors, has poor training stability, and its computational complexity increases with the square of the input resolution, making it difficult to efficiently process high-resolution images.

CoAtNet [24] is a combined model of Convolution and Attention. It captures local and global information of input data by introducing depthwise separable convolution (MBConv) and relative attention mechanism (Rel-attention). MBConv improves the generalization performance of the model under small sample conditions through the inductive bias of the convolutional network; Rel-attention uses relative position encoding to make up for the shortcomings of CNN and Transformer in processing position information. CoAtNet combines convolution operations with self-attention mechanisms into a basic computational unit and vertically stacks multiple computational units in an organized manner to construct a complete network architecture.

In this model, the convolution kernel operates as a fixed filter, making it well-suited for capturing translation-invariant features. In contrast, the self-attention mechanism enriches the model’s representational capacity and ability to model complex spatial dependencies through the incorporation of relative positional encoding. An effective design is achieved by integrating the convolution layer with an adaptive attention matrix—combining a static, globally applied convolution kernel with a dynamically updated attention matrix, applied before and after the softmax normalization. This hybrid approach capitalizes on the strengths of both operations. Equations (1) and (2) describe the stages prior to and following the softmax operation, respectively.

(1) $$y_{i}^{post}=\sum_{j\in G}\left(\frac{exp\left(x_{i}^{T}x_{j}\right)}{\sum_{k\in G}exp\left(x_{i}^{T}x_{k}\right)}+\omega_{i\text{-}j}\right)x_{j}$$

(2) $$y_{i}^{pre}=\sum_{j\in G}\frac{exp\left(x_{i}^{T}x_{j}+\omega_{i\text{-}j}\right)}{\sum_{k\in G}exp\left(x_{i}^{T}x_{k}+\omega_{i\text{-}k}\right)}x_{j}$$

where $i=(x_{i},y_{i})$, $j=(x_{j},y_{j})$, $k=\left(x_{k},y_{k}\right)$ represent specific coordinate positions in the global spatial space G, $\omega_{i\text{-}j}$ represents the weight matrix of position $\left(i\text{-}j\right)$, and G represents the global spatial space.

After combining the advantages of convolutional layers and self-attention layers, the network constructs a complete architecture by stacking. Due to the complexity of global context calculation, directly applying the relative attention mechanism will result in slow calculation. Therefore, it is necessary to downsample the feature map to reduce the spatial size and then use the global relative attention mechanism to enhance the feature representation. CoAtNet designed four model variants: C-C-C-C, C-C-C-T, C-C-T-T, and C-T-T-T (C represents the convolutional layer and T represents the self-attention layer), and compared them with the VIT [25] (Vision Transformer) model. The model capability and generalization ability results are shown in Equations (3) and (4).

(3) CCTT≈CTTT>VIT>CCCT>CCCC

(4) CCCC≈CCCT≥CCTT>CTTT≥VIT

**Figure 5:**
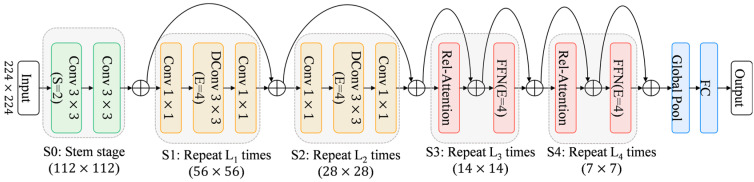
Architecture diagram of CoAtNet network.

It can be seen that simply adding Transformer modules does not necessarily improve model performance, whereas hybrid stacked structures such as C-C-T-T and C-T-T-T can more effectively enhance the model’s expressive capability. Considering generalization performance, the performance of C-C-T-T is comparable to that of C-C-C-T. Therefore, CoAtNet ultimately adopts the C-C-T-T structure to balance model performance and generalization ability. This architecture effectively enhances the overall performance through the reasonable configuration of convolutional layers and Transformer layers. In the S0 stage, two 3×3 convolutional layers are used for preliminary downsampling; in the S1 and S2 stages, convolutional modules are stacked to strengthen local feature learning; in the S3 and S4 stages, Transformer modules are introduced to capture long-range dependencies and global semantic information, and finally classification output is achieved through global average pooling and fully connected layers. In order to deal with the problems of gradient disappearance and explosion, the model introduces residual connections [26] in each submodule from S1 to S4 to promote gradient propagation and improve the training stability, convergence speed and generalization ability of the network. The architecture diagram of the CoAtNet network is shown in Figure 5.

#### 2.3.3. Fused-MBConv Module and MBConv Module

In CAM-UNet, the encoder’s S1 stage employs the Fused-MBConv module, while the S2 stage incorporates an enhanced MBConv block, enabling efficient extraction of multi-scale features. This configuration effectively enhances the network’s ability to extract complex textures and semantic features and significantly improves the model’s performance in fine-grained target recognition, precise boundary positioning, and adaptation to dynamic environments.

**Figure 6:**
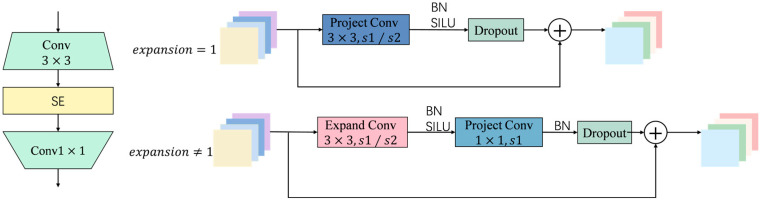
The Fused-MBConv module.

The Fused-MBConv module was first proposed in EfficientNetV2 as an improvement to the traditional MBConv architecture. Unlike MBConv, which performs 1×1 dimension-increasing convolutions and 3×3 depthwise separable convolutions in a stepwise manner, Fused-MBConv combines the dimension-increasing and spatial feature extraction processes into a single standard convolution operation, simplifying the network structure and significantly improving computational efficiency and representation capabilities in the early feature extraction stages. This is shown in Figure 6.

The Fused-MBConv module uses standard convolution at the front end to simultaneously complete channel expansion and spatial feature extraction, then introduces nonlinear activation functions to enhance expression capabilities, and finally achieves dimensionality reduction and restores the number of channels through point-by-point convolution. This structure has significant advantages in shallow feature extraction. It avoids the information loss that may be caused by deep separable convolution in low-level feature extraction and effectively improves the modeling capabilities of texture edges, small-scale targets and complex backgrounds while maintaining low computational overhead. It is particularly suitable for the recognition of high-frequency disturbances and small obstacles in dynamic water surfaces.

**Figure 7:**
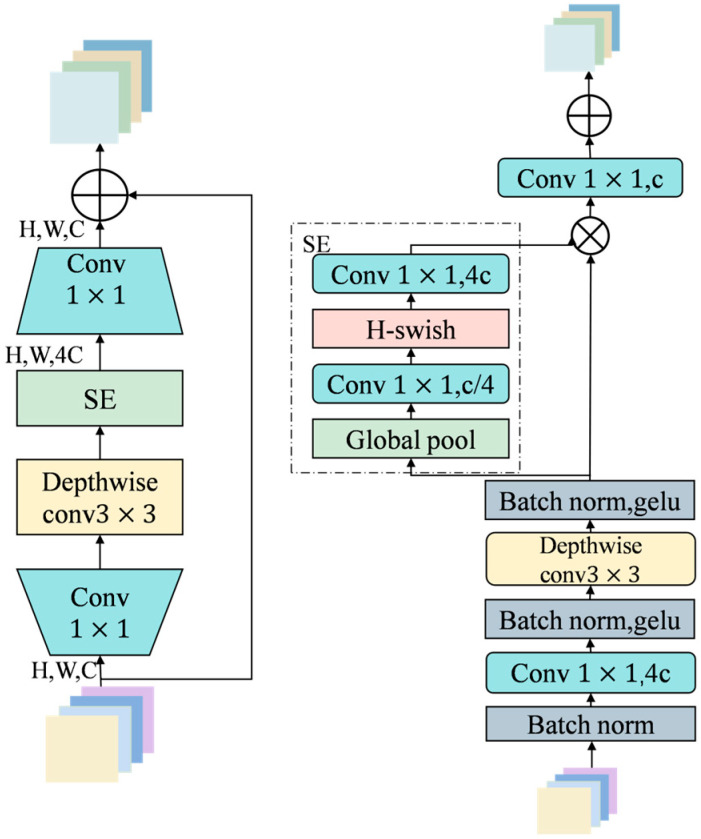
The Mbconv module.

As the network deepens, the MBConv [27] module is retained in the S2 stage. It adopts an inverted bottleneck structure consisting of 1×1 dimensionality increase convolution, 3×3 depth convolution, SE attention mechanism and 1×1 dimensionality reduction convolution, as shown in Figure 7. After the dimensionality increase, the structure performs deep convolution in high-dimensional space, which helps to more fully extract spatial structural features and strengthen the response of key areas through channel attention. Deep convolution significantly reduces parameters and computation by processing channels independently and enhances semantic expression capabilities while maintaining model compactness.

**Figure 8:**
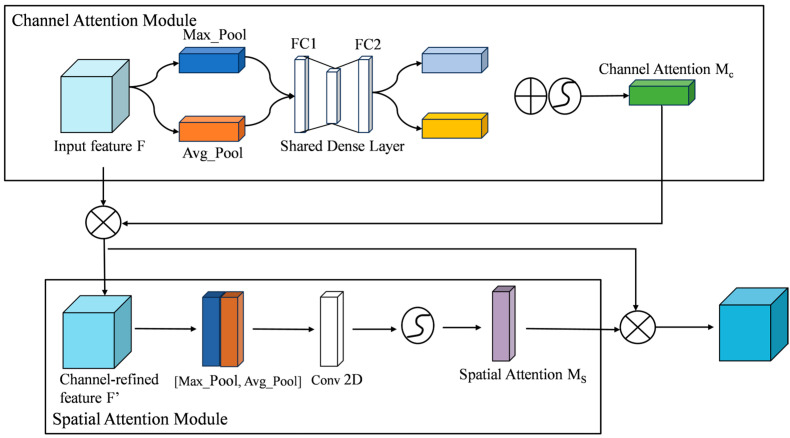
Structure of CBAM.

The traditional MBConv in MobileNetV3 uses the SE module to strengthen important features through global channel weighting, but it ignores spatial dimension information and has difficulty dealing with problems such as dynamic reflection and complex background interference. Therefore, in order to improve the model’s expression of important areas in complex water surface environments, this paper improves the attention mechanism in the MBConv module. The SE module is replaced with a CBAM that simultaneously models channel and spatial attention to achieve more refined feature focus and information guidance. CBAM [28] connects the channel attention and spatial attention mechanisms in series to achieve joint modeling of feature maps in both channel and spatial dimensions, significantly enhancing the model’s target perception ability in scenes with complex lighting, dynamic backgrounds, and partial occlusions. The channel attention submodule adopts a dual-branch structure of global average pooling and maximum pooling to extract global statistical information to evaluate the importance of each channel; the spatial attention submodule dynamically weights in the spatial dimension through convolution and activation functions to highlight key areas and structural edges. The CBAM architecture is shown in Figure 8.

#### 2.3.4. BiFormer Module

**Figure 9:**
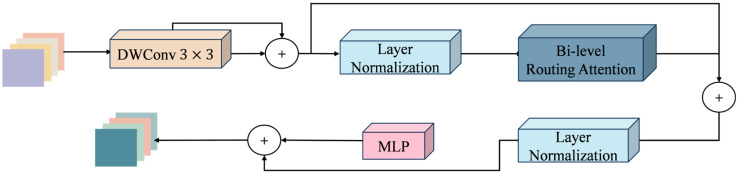
Structure diagram of the Biformer module.

In the encoder’s high-level semantic extraction stages (S3 and S4), this paper introduces the BiFormer module to enhance the model’s global modeling and detail representation capabilities in complex water surface scenes. The architecture of the Biformer module is shown in Figure 9.

BiFormer adopts a four-stage pyramid architecture. The initial stage generates primary features via overlapping patch embedding, while the subsequent second, third, and fourth stages progressively downsample the spatial resolution and expand the channel dimensions. Each stage is composed of several consecutive BiFormer blocks. Among the various module structures, depthwise separable convolution is primarily used to encode the spatial relative positional relationships, followed by a BRA (Bi-level Routing Attention) module and an MLP layer.

The BRA module applies a two-level routing strategy with dynamic sparse attention to filter out irrelevant key–value pairs, allowing the network to concentrate on salient regions. This design enhances the efficiency of feature extraction, strengthens multi-scale feature representation, and simultaneously reduces computational costs.

The 2D input feature map is divided into distinct, non-overlapping regions., each represented by feature vectors, from which query, key, and value tensors are obtained via separate linear transformations.

(5) $$Q=X^{r}W^{q},K=X^{r}W^{k},V=X^{r}W^{v}$$

where $W^{q},W^{k},W^{v}$ represents the projection weights of Q,K,V, respectively. Q,K,V represents query, key, and value. $X^{r}$ is the input feature of the attention module. The superscript r indicates that this is the feature tensor after regional division.

A feature averaging operation is performed within each non-overlapping region to aggregate local information and form region-level queries and keys.

(6) $$Q^{r}=\frac{\sum_{i=1}^{n}Q_{i}}{S^{2}},\;K^{r}=\frac{\sum_{i=1}^{n}K_{i}}{S^{2}}$$

where S is the length (height/width) of each non-overlapping area. $S^{2}$ represents the total number of tokens within an area.

Construct an adjacency matrix representing the relationships between regions.

(7) $$A^{r}=Q^{r}{\left(K^{r}\right)}^{T}$$

Only the top k connections of each region are kept to prune the dependency graph.

(8) $$I^{r}=topkIndex(A^{r})$$

where $I^{r}$ is the index matrix, k represents the maximum number of connections each region can establish with other regions.

Considering that each query requires access to the complete routing zone information, to improve memory access efficiency, the key–value pair tensors for the corresponding zones are pre-assembled.

(9) $$K^{g}=gather(K,I^{r}),\;V^{g}=gather(V,I^{r})$$

To improve the processing efficiency of the model under high-resolution or large-scale input, a more detailed token-to-token attention mechanism is applied to the pre-extracted key–value pairs.

(10) $$O=Attention(Q,K^{g},V^{g})+LCE(V)$$

**Figure 10:**
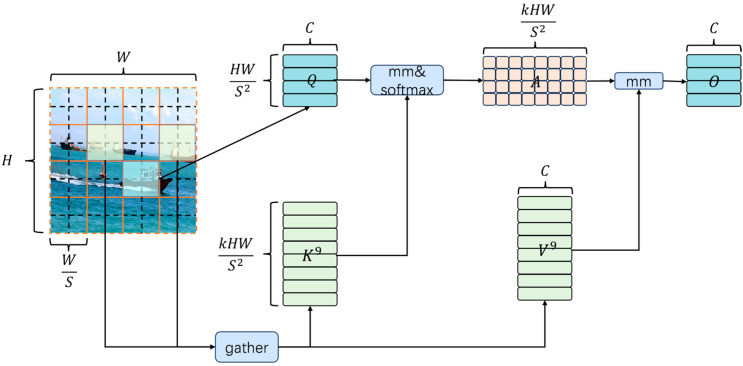
Structure of BRA.

The overall architecture of the BRA module is shown in the Figure 10 below.

#### 2.3.5. MSAA Module

The MSAA module consists of three main components: multi-scale fusion, spatial aggregation, and channel aggregation. Its design enhances the spatial awareness and channel representational capacity of feature maps, while keeping the additional computational cost minimal.

In the multi-scale fusion module, the input feature map $C_{1}\times H\times W$ is convolved for initial channel compression, producing an intermediate feature., resulting in an intermediate feature map $C_{2}\times H\times W$. This is then processed in parallel using three convolution kernels of different sizes: 3×3, 5×5, and 7×7 to extract feature information within the local and global receptive fields, respectively. These features, when concatenated in the channel dimension, fuse semantic representations from different scales, enhancing the model’s ability to model complex structures.

In the spatial attention aggregation module, the multi-scale fused feature map undergoes global average pooling to capture spatial context. A 7×7 convolution then generates a spatial attention map (the size of it is 1×H×W) that is normalized using a sigmoid activation function. The spatial attention map is applied element by element to the original feature map, highlighting spatially salient regions, suppressing background interference, and improving spatial discrimination.

The channel attention aggregation module first performs global average pooling on the input feature map to obtain a channel description vector which size is $C_{1}\times 1\times 1$. This is then generated through two channel-by-channel 1×1 convolutions (the first of which uses a ReLU activation function). This channel attention map is then multiplied channel-by-channel with the spatially weighted feature map to enhance salient channels and suppress redundant channels.

The enhanced features are resized using a 1×1 convolution and then added to the original input feature map using a residual connection, achieving a fusion of preserved original information and enhanced semantics.

**Figure 11:**
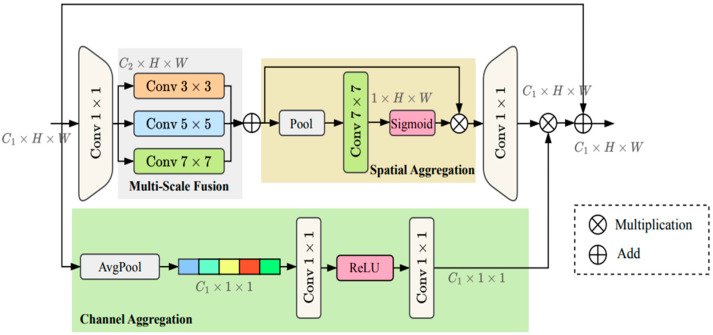
Structure diagram of MSAA module.

The MSAA module is shown in Figure 11. It enhances spatial position sensitivity and channel selectivity and is capable of addressing the perception challenges faced by convolutional networks in complex backgrounds and multi-scale object scenes. This enables its features to be more semantically rich and spatially aligned. It can provide more precise segmentation boundaries and more reliable small object detection for the decoder stage.

#### 2.3.6. U-Net Decoder

The decoder stage employs the U-Net decoder architecture [29]. In complex aquatic environments, as the encoding path abstracts higher-level semantics, spatial details gradually diminish. Accurately depicting irregular obstacles and detecting small targets are of crucial importance. The symmetrical U-Net structure and its skip connection mechanism can bridge the gap between semantic abstraction and spatial accuracy, effectively reversing the information loss of the encoder.

The high-level semantic features of the MSAA module are used as the input for the decoding stage, and the transposed convolutional layer restores the spatial resolution through progressive upsampling. Unlike traditional upsampling methods, the transposed convolution is learnable and can reconstruct the spatial structure more accurately.

One of the key features of this design is the skip connection, which combines the feature maps of the encoder with the corresponding decoder layers. This process injects the high-resolution spatial details from the early encoding stage into the decoding process, significantly improving the boundary accuracy. Additionally, it performs multi-scale feature fusion by combining deep, semantically rich features with shallow, spatially precise features, thereby enhancing the accuracy of target detection at different scales. It also supports gradient flow during the training process, which helps overcome the problem of gradient vanishing and ensures effective learning of the entire network.

After upsampling, by connecting each layer with the corresponding feature map in the encoder, the key spatial details and edge information are retained, thereby improving the accuracy and stability of the segmentation results. Then, the fused feature maps are refined using convolution and ReLU activation functions, which not only maintain the structural integrity of the decoder but also its interpretability. The symmetric architecture and skip connections of the U-Net decoder effectively utilize multi-scale context, successfully balancing deep semantic understanding with shallow spatial information. The segmentation prediction is both semantically accurate and spatially precise, meeting the high-performance requirements for analyzing complex aquatic environments.

## 3. Results and Discussion

### 3.1. Evaluation Metrics

This study evaluates the model’s performance using several metrics, including Mean Intersection over Union (mIoU), F1 score, Params, and FLOPs. A detailed explanation of each metric is provided below.

Using the confusion matrix, predictions are classified as positive or negative, with each prediction being either correct or incorrect. This results in four categories for segmentation outcomes: True Positives (TPs), True Negatives (TNs), False Positives (FPs), and False Negatives (FNs).

Mean Intersection over Union (mIoU) is a commonly used metric for evaluating semantic segmentation models. It measures the overlap between the predicted segmentation and the ground truth annotations, offering a comprehensive assessment of the model’s performance across multiple classes. To calculate mIoU, the Intersection over Union (IoU) is computed for each class individually, and the average is then taken. Specifically, IoU is the ratio of the intersection to the union of the predicted region and the corresponding ground truth region. Higher IoU values indicate a closer alignment between the model’s segmentation and the true labels. The formula is given as follows:

(11) $$IoU=\frac{TP}{FP+FN+TP}$$

(12) $$MIoU=\frac{\sum_{i=1}^{n}IoU_{i}}{n}$$

The F1 score serves as a balanced metric for evaluating binary classification performance by computing the harmonic mean of Precision and Recall. Its formula is presented in Equation (15). The F1 score ranges from 0 to 1, where values closer to 1 signify superior model accuracy.

(13) $$Recall=\frac{TP}{TP+FN}$$

(14) $$Precision=\frac{TP}{TP+FP}$$

(15) $$F1=\frac{2\times Precision\times Recall}{Precision+Recall}$$

Params refer to the total number of trainable parameters in a model. This metric captures the model’s size and its spatial complexity. Generally, a higher parameter count indicates a more complex model, which imposes a direct impact on the memory and computational power required for both training and inference.

(16) $$Params=\left(k_{h}\times k_{w}\times c_{in}+1\right)\times c_{out}$$

where $k_{h}$ represents the kernel height, $k_{w}$ represents the kernel width, $c_{in}$ represents the number of input channels, $c_{out}$ represents the number of output channels, and the +1 accounts for the bias term.

FLOPs measure the total number of floating point operations a model performs, indicating its computational cost or time complexity. Since each addition or multiplication counts as one FLOP, a higher FLOP count generally implies a greater demand for computational resources.

(17) $$FLOPs=2\times k_{h}\times k_{w}\times c_{in}\times H_{out}\times W_{out}\times c_{out}$$

where $H_{out}$ represents the height of the output feature map, $W_{out}$ represents the width of the output feature map, and multiplication by 2 indicates that a convolution operation includes one multiplication and one addition.

### 3.2. Ablation Experiments

To verify the effectiveness of the key modules proposed in this paper in the task of water surface feasible domain and obstacle segmentation, we conducted a series of ablation experiments on a self-constructed dataset. The basic model uses CoAtNet as the backbone network and U-Net as the decoder. IoU1 and IoU2 represent the Intersection over Union (IoU) of the obstacle and feasible domain categories, respectively. The experimental results are shown in Table 2.

**Figure 12:**
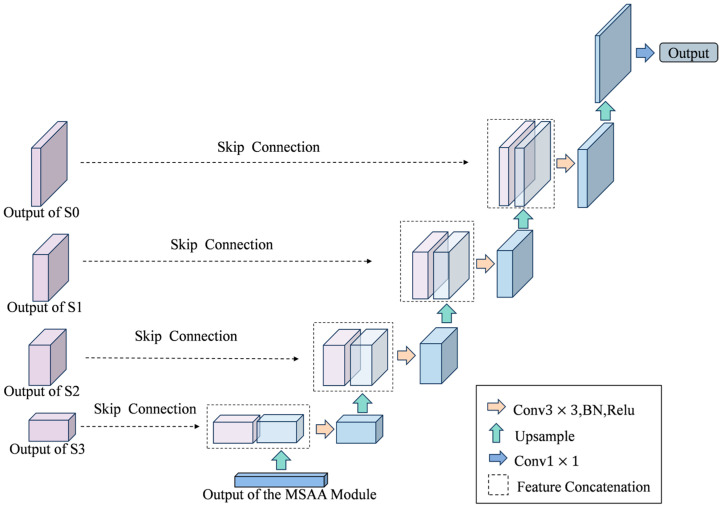
Structure diagram of U-Net decoder.

The basic model without other structural optimizations, the model achieves mIoU of 89.31%. However, its IoU1 performance in obstacle areas is relatively weak, at only 84.23%, indicating its limited ability to represent boundary structures and small objects. Figure 12 shows that the CoAtNet-Base model performs poorly in segmentation, with issues such as blurred boundaries around obstacles and feasible regions, and errors in obstacle segmentation.

In model (a), the Fused-MBConv and CBAM (model b) were introduced. The mIoU increased from 89.31% to 92.37%, and the obstacle IoU1 rose from 84.23% to 88.91%. This indicates that by improving local feature extraction and spatial attention, making it more efficient in capturing texture and edge details, it greatly contributes to enhancing the model performance.

The base of model (b) has enhanced the local capabilities. Based on this, we introduced the BiFormer module (model d) at the advanced semantic stage (S3/S4). This version achieved an mIoU of 94.15%, and its performance surpassed that of model (c)—including local enhancement and the MSAA module (93.1%), while requiring fewer computing resources. This indicates that using BiFormer for global context modeling at the high semantic stage brings more benefits than adding the MSAA module, providing significant advantages in terms of efficiency and performance.

**Figure 13:**
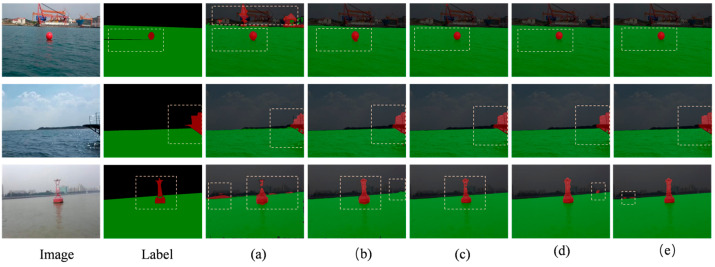
Comparison of ablation experiment results.

When all the modules are integrated into the complete model (e), the mIoU reaches 95.15%. The improvement compared to model (d) confirms that the combination of MSAA with a powerful local–global representation is very valuable. The comparison of the experimental results can be seen in Figure 13.

### 3.3. Comparative Experiments

To evaluate the performance of the proposed CAM-UNet model, we compared it with several advanced image segmentation models under the same experimental conditions. These included the classic U-Net [29], the U-Net variant with the enhanced CoatNet backbone (U-Net (CoAtNet)), DeepLabv3+ [30], DANet [31], SegFormer-B1 [19], and SeaFormer-B [32], along with the domain-specific model WaSRNet [33].

U-Net is renowned for its simplicity and powerful boundary restoration capabilities and is the preferred choice for many segmentation tasks. The U-Net (CoatNet) version improves the traditional design by using the powerful CoatNet backbone network. DeepLabv3+ introduces the Atrous Spatial Pyramid Pooling (ASPP) module, enhancing multi-scale context modeling and achieving high accuracy in semantic segmentation. DANet combines channel-level and spatial attention modules within the standard CNN framework, improving the model’s focus on key areas and enhancing object recognition in complex scenes. SegFormer is a relatively new lightweight Transformer-based model that effectively combines global context modeling with fast decoding processes, providing excellent inference speed without sacrificing accuracy.

SegFormer is a Transformer-based model that effectively combines global context modeling with fast decoding processes, providing excellent inference speed. SeaFormer balances performance and computational efficiency through lightweight CNN and attention mechanisms, enhancing feature representation. It is particularly suitable for environments with limited computing resources. WaSRNet is a model for the marine segmentation field. Although it has high computational requirements, it is good at identifying marine objects and is a key benchmark in this field.

All models were trained and tested on the self-built WSM USV dataset. The experimental results are shown in Table 3.

U-Net demonstrates the reliability of its simple architecture but also reveals limitations in computational efficiency. U-Net (CoatNet) significantly improves efficiency while maintaining model accuracy by introducing a more advanced backbone network, showcasing the impact of backbone network choice on model performance. DeepLabv3+ improves segmentation accuracy through multi-scale context modeling, but this increases the number of parameters and reduces efficiency. DANet achieves excellent performance using a complex dual-attention mechanism, but this also incurs very high computational costs. In contrast, SegFormer-B1 achieves a better balance between complexity and performance with its innovative hierarchical Transformer design. SeaFormer-B, as a lightweight model, offers both efficiency and performance. WaSRNet performs well in ocean segmentation, but its high computational cost limits its practical application in real-world scenarios.

CAM-UNet integrates several key modules: The encoder combines Fused-MBConv and CBAM to enhance shallow feature extraction and improve perception of important areas; the BiFormer module at a higher semantic level strengthens long-range dependency modeling and semantic representation; the MSAA module effectively integrates multi-scale semantics and spatial information; the decoder maintains the skip connections of U-Net style to retain spatial details during upsampling. These designs give CAM-UNet greater adaptability and stability in complex water surface scenarios, and can effectively handle small obstacles, blurred edges, etc.

Although the number of parameters in CAM-UNet is greater than that of some lightweight models, in scenarios where maritime navigation safety is of utmost importance, we will prioritize accuracy. Despite the increase in computational complexity, this model has achieved a significant improvement in accuracy, which is of great help in ensuring the perception system of unmanned surface vessels (USVs) operating in challenging marine environments.

**Figure 14:**
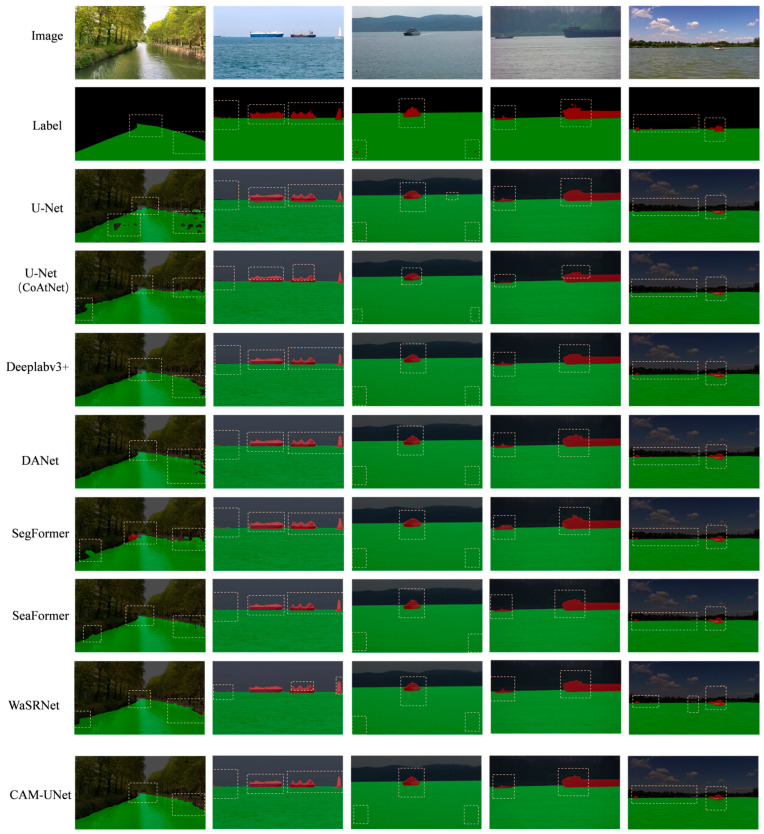
Comparison of experimental results.

Figure 14 presents semantic segmentation results for five representative images across different methods. The visual comparisons clearly show that CAM-UNet outperforms other models in various challenging water scenes, accurately detecting small obstacles and fine shoreline details with clear boundaries and well-preserved structures.

Having established CAM-UNet’s effectiveness on the specialized WSM USV dataset, we now assess its generalizability on public benchmarks. Experiments were conducted on the MaSTr1325.

The MaSTr1325 dataset is widely used in the field of unmanned surface vessels (USVs) in the ocean. This dataset can meet the obstacle detection requirements of small coastal USVs, and it contains 1325 images captured on-site. These photos were taken during a two-year coastal patrol mission by a USV, and each image includes pixel-level semantic annotations of three categories: sky, ocean, and obstacle. The evaluation uses three IoU metrics: IoU1 (sky), IoU2 (ocean), and IoU3 (obstacle). The experimental results are shown in Table 4.

Our model achieved an mIoU of 98.48% on the dataset, surpassing all other methods, and obtained good IoU in the three categories of sky, ocean, and obstacles. In the crucial “obstacle” category, CAM-UNet achieved an IoU of 96.37%, significantly outperforming other models. This result demonstrates its ability to accurately identify obstacles in complex marine environments. Compared with WaSRNet, which is specifically designed for marine applications, CAM-UNet not only achieved higher segmentation accuracy but also maintained strong model efficiency, showcasing excellent overall performance. These results indicate that CAM-UNet not only performs well on the dedicated local dataset but also impressively performs in public benchmark tests. It has been proven to be highly competitive and adaptable, capable of handling diverse marine environments and task requirements.

### 3.4. Robustness Evaluation

To further assess the model’s robustness, we subjected the test set to Gaussian noise, color jitter, and image blurring, creating three sub-test sets that simulate more complex surface disturbance conditions. We then performed semantic segmentation experiments on these sets using CAM-UNet, SeaFormer-B, and WaSRNet, with mean Intersection over Union (mIoU) as the primary evaluation metric.

**Figure 15:**
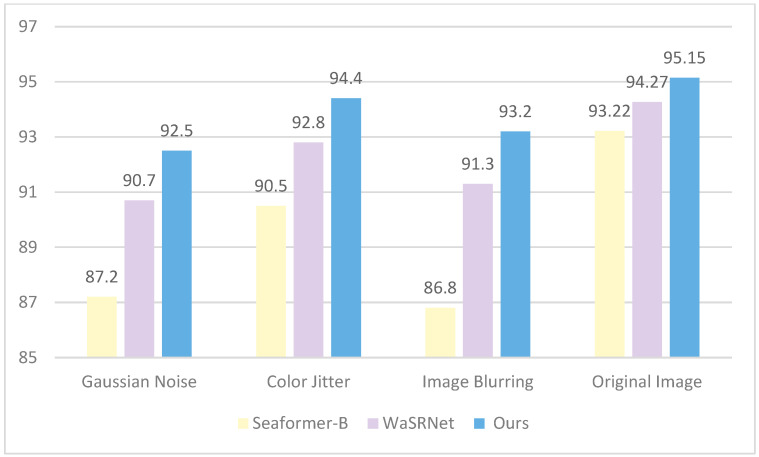
Robustness experiment results.

The experimental results shown in Figure 15 indicate that CAM-UNet outperforms other models in terms of stability under various disturbances. It maintains the highest mIoU across all test conditions, demonstrating particularly strong resilience to Gaussian noise and image blurring. In contrast, CAM-UNet excels in maintaining stability in disturbed environments while also preserving high accuracy on original images, thanks to its effective feature extraction and fusion mechanisms. While WaSRNet performs well on original images, its significant drop in performance under disturbances highlights its sensitivity to changes in image quality. These findings confirm that CAM-UNet’s architecture offers enhanced reliability in challenging maritime environments, where image quality can often be compromised.

## 4. Conclusions

The proposed CAM-UNet has performed exceptionally well in semantic segmentation tasks in complex water environments. This model is constructed based on the improved CoAtNet backbone network and uses U-Net as the decoder. In the encoder, it integrates Fused-MBConv, CBAM, and BiFormer modules, and uses the MSAA module for multi-scale feature fusion. Experimental results show that CAM-UNet outperforms mainstream models such as DeepLabV3+, SegFormer, and SeaFormer in multiple key evaluation metrics. It excels in boundary restoration and small object detection. However, while the model can be deployed on most modern USV embedded systems, its implementation on resource-constrained platforms may require additional optimization. Future research will focus on optimizing model performance from multiple aspects, including fusing multi-source image information (such as visible light and infrared) to enhance stability in low-light and high-interference environments, expanding the model’s temporal modeling capabilities to support video-level semantic segmentation tasks, and compressing the model size through lightweight design and efficient feature extraction strategies to improve its deployment efficiency and application value in practical scenarios such as intelligent navigation, water monitoring, and port management.

## Figures and Tables

**Table 1:** Experimental equipment.

Software and Hardware Configuration	Version Model
GPU	NVIDIA GeForce RTX 4070 (12 GB VRAM)
CPU	Intel Core i7-13700KF
Framework	PyTorch 1.13.0 + Python 3.8
Acceleration	CUDA 11.6, cuDNN 8.4

**Table 2:** Results of the ablation experiment.

Sequence Number	CoAtNet-Base	Fused-MBConv & CBAM	BiFormer	+MSAA	FLOPs	Params (M)	mIoU (%)	IoU1	IoU2
(a)	✓				32.5	29.8	89.31	84.23	94.39
(b)	✓	✓			33.2	30.5	92.37	88.91	95.83
(c)	✓	✓		✓	41.5	35.3	93.1	89.5	96.7
(d)	✓	✓	✓		36.8	33.1	94.15	91.44	96.86
(e)	✓	✓	✓	✓	42.3	37.8	95.15	92.86	97.44

**Table 3:** Results of comparative experiments on custom dataset.

Method	Params (M)	FLOPs	mIoU (%)	F1-Score (%)
U-Net	31.2	65.2	88.42	88.7
U-Net (CoatNet)	29.8	32.5	89.31	89.6
DeepLabv3+	41.3	64.3	91.04	91.5
DANet	67.4	86.6	92.13	92.6
SegFormer-B1	13.7	16.8	90.77	91.1
SeaFormer-B	8.2	9.3	93.22	93.8
WaSRNet	70.8	87.2	94.27	94.8
CAM-UNet (Ours)	37.8	42.3	95.15	95.3

**Table 4:** Results of comparative experiments on MaSTr1325.

Method	mIoU (%)	IoU1 (%)	IoU2 (%)	IoU3 (%)
U-Net	97.57	99.21	98.86	94.65
DeepLabV3+	96.73	99.09	98.36	92.75
SegFormer-B1	97.07	98.55	98.92	93.73
SeaFormer-B	97.90	99.42	99.33	94.95
WaSRNet	98.27	99.54	99.39	95.87
CAM-UNet (Ours)	98.48	99.66	99.42	96.37

## Data Availability

The datasets presented in this article are not readily available because the data are part of an ongoing study. Requests to access the datasets should be directed to nfugxy@njfu.edu.cn.
